# Supplementary figures and images for: The impact of COVID-19 pandemic on ridesourcing services differed between small towns and large cities
Source: PLoS One. 2022 Oct 14;17(10):e0275714. doi: 10.1371/journal.pone.0275714 (PMC9565726; doi:10.1371/journal.pone.0275714)

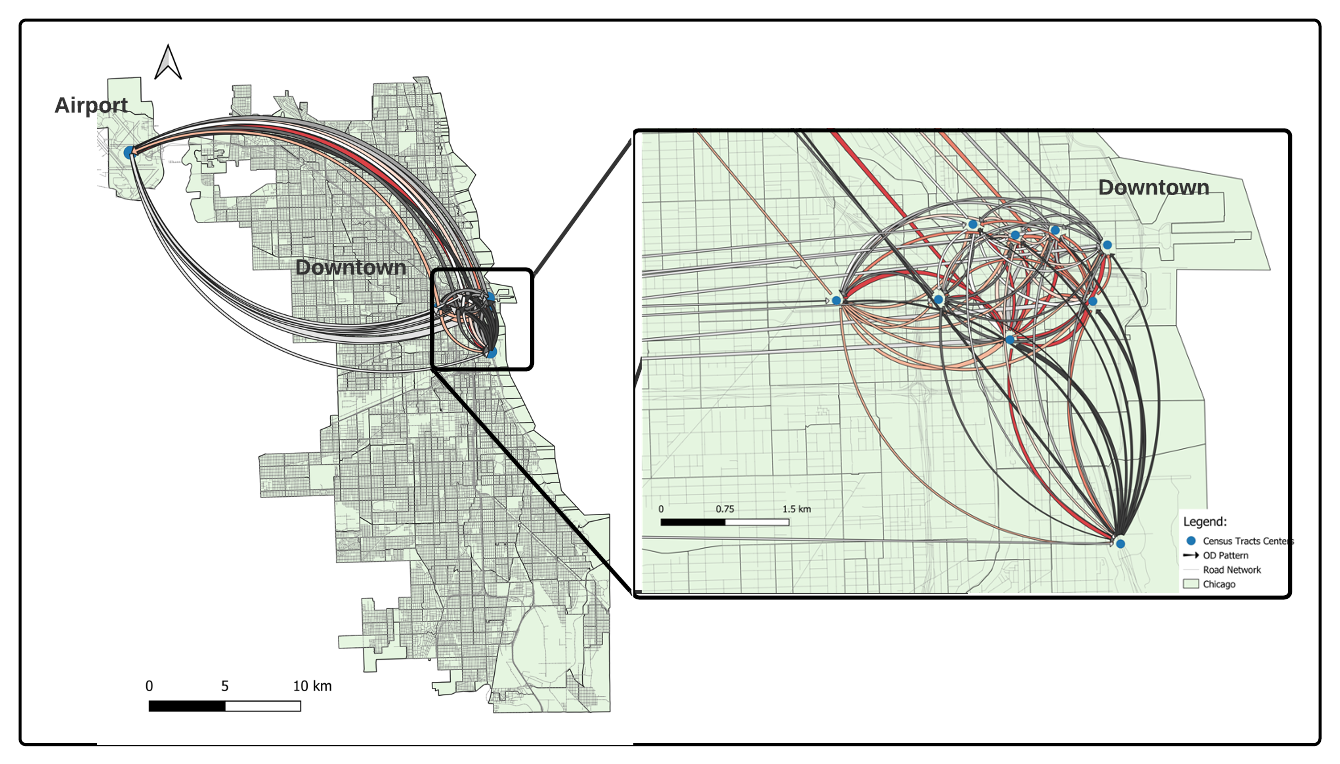

Supplement: S1 Fig — As during the pandemic, high-demand OD pairs were in the downtown area and between the downtown and O’Hare International Airport. The pre-pandemic trip data in Innisfil did not have the required information to develop the OD flow patterns. However, the ridesourcing trip density maps developed by Sweet et al. [62] for the time period from May 2017 to February 2020, showed that the Innisfil’s central area had the highest pick-up and drop-off density, which is similar to the patterns observed during the pandemic. (TIF) [file pone.0275714.s001.tif]
