# Supplementary material for: The impact of COVID-19 pandemic on ridesourcing services differed between small towns and large cities
Source: PLoS One. 2022 Oct 14;17(10):e0275714. doi: 10.1371/journal.pone.0275714 (PMC9565726; doi:10.1371/journal.pone.0275714)
Supplement: S1 Table — (DOCX) [file pone.0275714.s002.docx]

| **Hyperparameters** | **Model** | | | |
| --- | --- | --- | --- | --- |
|  | **Percent Reduction in Daily Demand** | | **Direct Demand** | |
|  | **Town of Innisfil** | **City of Chicago** | **Town of Innisfil** | **City of Chicago** |
| Criterion | Mean Squared Error | Mean Squared Error | Mean Absolute Error | Mean Squared Error |
| Maximum Depth | 20 | 10 | 50 | None |
| Maximum Features | Log2 (No. of Features) | Log2 (No. of Features) | Log2 (No. of Features) | No. of Features |
| Minimum Sample Leaf | 1 | (0.0028 x Sample Size) | 1 | 1 |
| Minimum Sample Split | 2 | (0.0227 x Sample Size) | 2 | 2 |
| Number of Estimators | 100 | 100 | 150 | 100 |
| Maximum Leaf Nodes | 100 | 100 | None (Unlimited) | None (Unlimited) |
| Bootstrap | True | False | True | True |
