# Supplementary material for: The impact of COVID-19 pandemic on ridesourcing services differed between small towns and large cities
Source: PLoS One. 2022 Oct 14;17(10):e0275714. doi: 10.1371/journal.pone.0275714 (PMC9565726; doi:10.1371/journal.pone.0275714)
Supplement: S2 Table — Across all performance metrics, the models in Chicago had a higher predictive accuracy than those in Innisfil. This is most likely because Chicago’s datasets contained more samples. (DOCX) [file pone.0275714.s003.docx]

| **Performance Metric** | **Model** | | | |
| --- | --- | --- | --- | --- |
|  | **Percent Reduction in Daily Demand** | | **Direct Demand** | |
|  | **Town of Innisfil** | **City of Chicago** | **Town of Innisfil** | **City of Chicago** |
| Mean Absolute Error (MAE) | 0.09 | 0.03 | 1.24 | 0.81 |
| Mean Squared Error (MSE) | 0.015 | 0.002 | 3.49 | 4.52 |
| Root Mean Squared Error (RMSE) | 0.12 | 0.042 | 1.87 | 2.13 |
| Coefficient of Determination (R^2^) | 0.59 | 0.93 | 0.81 | 0.85 |
