# Supplementary material for: The impact of COVID-19 pandemic on ridesourcing services differed between small towns and large cities
Source: PLoS One. 2022 Oct 14;17(10):e0275714. doi: 10.1371/journal.pone.0275714 (PMC9565726; doi:10.1371/journal.pone.0275714)
Supplement: Attachment — Submitted filename: Response_to_Reviewers.pdf [file pone.0275714.s004.pdf]

The impact of COVID-19 pandemic on ridesourcing services differed between small towns and large cities

Nael Alsaleh, Bilal Farooq

Response to the reviewers
August 8, 2022

We would like to thank the editor and the reviewers for their careful review, constructive recommendations and positive and encouraging comments. We appreciate the time and effort that the reviewers dedicated to provide valuable feedback on our manuscript. In the following, we have listed the reviewers' comments, followed by our point-to-point responses in the gray box. In this document, texts in *"italic"* and blue refer to manuscript texts of the revised version of our paper that are directly quoted here.

The minimal dataset underlying the results described in our paper, including all the data needed to reproduce the figures, values behind the descriptive statistics, and code to generate the demand models, is available on a public repository at:
https://github.com/LiTrans/COVID-19-Impacts-on-Ridesourcing-Services.git

The repository also contains a URL to access the full operational data for the ridesourcing services in Chicago, and the contact information for requesting operational data for Uber Transit in Innisfil.

We would also like to bring to the attention of the editor and reviewers that we have modified our affiliation to reflect the new name of the university. The new name is Toronto Metropolitan University.

Journal Requirements

1. We notice that your manuscript file was uploaded on March 17, 2022. Please can you upload the latest version of your revised manuscript as the main article file, ensuring that does not contain any tracked changes or highlighting. This will be used in the production process if your manuscript is accepted. Please follow this link for more information:
http://blogs.PLOS.org/everyone/2011/05/10/how-to-submit-your-revised-manuscript/

Response:

Thank you for the comment. We have now uploaded the latest version of our manuscript.

2. We note that your manuscript is not formatted using one of PLOS ONE's accepted file types. Please reattach your manuscript as one of the following file types: .doc, .docx, .rtf, or .tex (accompanied by a .pdf).
If your submission was prepared in LaTex, please submit your manuscript file in PDF format and attach your .tex file as "other."

Response:

We have now provided the (.tex) file along with the manuscript.

3. Please upload a copy of Figure 4 which you refer to in your text. Or if the figure is no longer to be included as part of the submission please remove all reference to it within the text.

Response:

We apologize for this mistake. We have now provided all Figures cited in the text.

4. As you may already be aware, PLOS journals require authors to make all data underlying the findings described in their manuscript fully available without restriction at the time of publication https://journals.plos.org/plosone/s/data-availability. However, we do allow for rare exceptions to this strict requirement in cases where there are ethical or legal considerations with respect to the public sharing of the underlying data.
Please also note that the data we ask authors to share is not necessarily the full and/or raw data set collected in the course of their research, but rather the "minimal data set", which we define as consisting of the data set used to reach the conclusions drawn

in the manuscript with related metadata and methods, and any additional data required to replicate the reported study findings in their entirety. Depending on the nature of your study, this can include any or all of the following:

- **The values behind the means, standard deviations and other measures reported;**
- **The values used to build graphs;**
- **The points extracted from images for analysis.**

We note that you have indicated that data from this study are unavailable due to unacceptable data access restrictions for submissions to PLOS ONE.
Please note that PLOS journals will not consider manuscripts for which the following factors influence authors' ability to share data:

a) Authors will not share data because of personal interests, such as patents or potential future publications.
b) The conclusions depend solely on the analysis of proprietary data. We consider proprietary data to be data owned by individuals, organizations, funders, institutions, commercial interests, or other parties that the data owners will not share. If proprietary data are used and cannot be accessed by others in the same manner by which the authors obtained them, the manuscript must include an analysis of publicly available data that validates the study's conclusions so that others can reproduce the analysis and build on the study's findings.
Please be aware we will not proceed with your submission if you are unable provide publicly available minimal data for your study. Please see our full Data Availability guidelines here: https://journals.plos.org/plosone/s/data-availability. If you are able to provide the minimal data for your study, please address the following relevant prompt in an updated Data Availability statement with your submission:
a) If there are ethical or legal restrictions on sharing a de-identified data set, please explain them in detail (e.g., data contain potentially identifying or sensitive patient information) and who has imposed them (e.g., a Research Ethics Committee or Institutional Review Board, etc.). Please provide non-author contact information (phone/email/ hyperlink) for a data access committee, ethics committee, or other institutional body to which data requests may be sent. If applicable, please also provide any necessary information which interested researchers would need when requesting access to data in order to obtain the minimal data set for your study.
b) If your minimal data were obtained from a third party, please explain how others can access or request the specific datasets related to your research. Confirm that others would be able to access or request these data in the same manner as the authors. Please also confirm that the authors did not have any special access or request privileges that others would not have.
c) If there are no restrictions, and your minimal data were not obtained from a third party, please upload the minimal anonymized data set necessary to replicate your study findings to a stable, public repository and provide us with the relevant URLs, DOIs, or

accession numbers. Please see http://www.bmj.com/content/340/bmj.c181. long for guidelines on how to de-identify and prepare clinical data for publication. For a list of recommended repositories, please see https://journals.plos.org/plosone/s/recommended-repositories.
You also have the option of uploading the data as Supporting Information files, but we would recommend depositing data directly to a data repository if possible.
We will update your Data Availability statement with the information you provide.

Response:

Thank you for providing us with the information. The minimal dataset underlying the results described in our paper, including all the data needed to reproduce the figures, values behind the descriptive statistics, and code to generate the demand models, is now available on a public repository at:
https://github.com/LiTrans/COVID-19-Impacts-on-Ridesourcing-Services.git
The repository also contains a URL to access the full operational data for the ridesourcing services in Chicago, and the contact information for requesting operational data for Uber Transit in Innisfil.

5. We note your response to the copyright query: “”Thank you for providing us the information. To the best of our understanding, we have not included any satellite images or used google software. The Figure you referred to was developed by us in a free and Open Source Geographic Information System (QGIS). The maps presented in this Figure, contain 4 layers, including the boundary shape files for both study areas, train stations’ location, central points, and OD patterns. The open access boundary shape files were obtained from Statistics Canada & the American Central Statistics Office. We developed the remaining layers using QGIS”
Thank you for letting us know that Figure 3 was created with QGIS. Please note the following from QGIS’s website: “If not stated otherwise, all content is licensed under Creative Commons Attribution-ShareAlike 3.0 licence (CC BY-SA)”. Please note that as PLOS the CC BY 4.0 license https://creativecommons.org/licenses/by/4.0/ meaning that all material on our website is freely available online, and any third party is permitted to access, download, copy, distribute, and use these materials in any way, even commercially, with proper attribution, CC BY-SA is incompatible.
Therefore, we kindly ask that you state in the figure caption that the figure is licensed under CC BY 4.0 license so we may proceed.

Response:

Thank you for providing us the information. We have now included this statement in the figure’s caption.

The caption of Figure 3 is now as follows:

"Origin-destination (OD) flow patterns for ridesourcing services during the pandemic in (a) Innisfil and (b) Chicago. In Innisfil, 52,126 ridesourcing trips were made from September 2020 till August 2021 covering 20 CTs, while more than 21 million trips were made in Chicago during the pandemic distributed over 803 CTs. Since it is hard to develop meaningful flow patterns for all OD pairs, we only display the flow patterns for the 10 most frequently used CTs in both locations. The OD flows shown in this figure represent 90% and 10% of the total trips in Innisfil and Chicago, respectively. The OD line color represents the origin of trips and the width reflects the flow strength. The wider the line is, the more trips the OD pair has. This figure was generated using the free and open-source software QGIS under the CC BY 4.0 license."

Reviewer 1

The authors investigated and compared factors influencing the use of RHA in the cities of Chicago, IL and Innisfil, ON during the COVID-19 pandemic using the random forest regression method. The results revealed the uniqueness of RHA trip characteristics in the big and the small cities and showed the different impacts of the COVID-19 pandemic on the use behaviors of RHA in these two cities. Their results can be useful for transport planners and policymakers to better understand how people were dependent on RHA and how the sensitivity of RHA use to the change in travel demand characteristics (e.g., due to the pandemic, policy, new lifestyle). However, several improvements are required before the manuscript can be published.

Reply: We would like to thank the reviewer for the constructive comments and suggestions.

Reviewer Point 1.1 — What's the point of comparing big and small cities, and more specifically Innisfil and Chicago which are vastly different, not only in their sizes but also in different countries as well as transportation system infrastructure and travel demand characteristics. It's no surprise that Covid would affect them in different ways. The analysis of Chicago data alone can potentially provide sufficient scientific contribution. How would data from the tiny Innisfil add to scientific knowledge? Why not the town of the same size in different countries, or different sizes in the same country? You need to provide more convincing justification of the choice of study areas.

Reply: Thank you for the comment. We agree that comparing different towns/cities with each other or even including more case studies can provide useful insights. However, the actual data for ridesourcing services is very limited and only provided under license in most cases. Additionally, the motivation for study was the need to understand and compare the impact of COVID-19 across urban areas within the same geographical region. Several factors governed the selection of the case studies, including a) the availability of ridesourcing data, b) the coverage of both the pre-pandemic and pandemic periods, and c) the location of both case studies within the same region. After gaining access to the actual ridesourcing data in the Town of Innisfil, the City of Chicago was selected as a second case study. To the best of our knowledge, the City of Chicago is the only case study, where a publicly available ride sourcing data satisfies the above-mentioned criteria.

The Town of Innisfil and the City of Chicago are located in North America, where the population is concentrated in the largest cities, while rural areas, are sparsely populated. The case studies have similar weather conditions, share some sociodemographic characteristics, such as percentage of males, percentage of working age group, and average household size, and have experienced various NPIs during the pandemic. On the flip side, as you have mentioned, they differ in the land area, population density, the use of ridesourcing services and their operating policies, as well as the severity of NPIs over time. In this study, we examined the impacts of the COVID-19 pandemic in

both two case studies and discussed how these differences have resulted in the COVID-19 pandemic having different effects.

The City of Chicago has a rich dataset that, as you stated, provides several insights on how the pandemic and its related characteristics have impacted ridesourcing services. Adding data from Innisfil, however, contributed to a better understanding of the similarities and differences in the demand for ridesourcing services among small towns (low-density area) and big cities (high-density area). As discussed in the results, most of the socioeconomic and sociodemographic characteristics, land use variables, COVID-19 related variables and NPIs, as well as trips characteristics had the same impact on the ridesourcing services in both locations. But the relative importance of these variables differed between the two case studies. Second, it contributed to a better understanding of the pandemic effects on the ridesourcing services, after linking the results to the characteristics of each case study.

We have added the summary of this discussion at the beginning of the "Data collection" as well as "Discussion and concluding remarks" sections.

- In the "Data collection" section:

"This study was motivated by the need to understand and compare COVID-19 pandemic impacts across urban areas within the same geographical region. The Town of Innisfil and the City of Chicago we chosen as case studies. Several factors governed the selection of these case studies, including a) the availability of ridesourcing data, b) the coverage of both the pre-pandemic and pandemic periods, and c) the location of both case studies within the same region (North America). The Town of Innisfil is located on the western shore of Lake Simcoe in Simcoe County, Ontario, with a population density of 139 people per square kilometer. According to the 2016 census data, the town has a population of 36,566 people, around 67% of whom are in the working age group (15 to 64 years old). The median household income (after-tax) in Innisfil is $57,846, which is higher than the national average of $48,895 [1]. On the other hand, the City of Chicago is located on the southwestern tip of Lake Michigan in Illinois and is considered among the largest cities in the United States, with a population density of 4,618 persons per square kilometer. The City of Chicago has a population of more than 2.7 million and almost 70% of the population belongs to the working age group. The median household income (after-taxes) in Chicago is $62,613, which is lower than the national average of $70,690 [2]."

- In the "Discussion and concluding remarks" section.

"The on-going COVID-19 pandemic has significantly impacted individual's mobility [24, 25]. Understanding these effects can help planners and policy makers to better anticipate the pandemic's future impacts, which may contribute both to recovery efforts and plan responses to additional waves of COVID-19 or other pandemics. Previous studies, however, have only considered the impacts of COVID-19 on ridesourcing services in early stages and have primarily focused on large cities. This is mainly due to the apparent lack of readily available ridesourcing data. In this study, we examined and compared the impact of the COVID-19 pandemic on ridesourcing services in small towns and large cities using actual data from the Town of Innisfil, Ontario, and the City of Chicago, Illinois. The main factors that governed the selection of these case studies were: a) the availability of ridesourcing data, b) the coverage of both the pre-pandemic and pandemic periods, and c) the location of both case

studies within the same region (North America). The two case studies are located in North America, have similar weather conditions, share some sociodemographic characteristics, such as percentage of males, percentage of working age group, and average household size, and have experienced various NPIs during the pandemic. On the flip side, they differ in the land area, population density, the use of ridesourcing services and their operating policies, as well as the severity of NPIs over time."

Reviewer Point 1.2 — The methodology section needs elaboration. The diagram in Figure 1 should be explained in more detail. Also check for typos. Explanations of specific techniques, including Random forest regression, Bayesian optimization, and technical measurements (e.g., SHAP value, stringency index) are needed. Some equations and detailed descriptions of definition of dependent variables can be helpful.

Reply: Thank you for the comment, and we apologize for the typos in Figure 1. We have revised the methodology section and made the following changes:

- Adding a new subsection "Methods", where we explain the main technical measurements and techniques used in this study, including: Stringency Index, Random Forest, Bayesian Optimization, and SHAP Analysis.
- Using equations to explain the dependent variables of both the percent reduction in daily demand and the direct demand models (In "Data analysis and modelling" section).
- Adding more information on how the techniques have been implemented in our study to the last paragraph of "Data analysis and modelling" section.
- Correcting the oversights in Figure 1.

"Methods

Stringency index

Oxford COVID-19 Government Response Tracker (OxCGRT) gathers publicly available data on 23 indicators of policy measures governments have taken to respond to the pandemic from more than 180 countries. There are eight indicators related to the government's containment and closure policies (C1-C8), eight indicators related to the health system policies (H1-H8), four indicators related to economic policies (E1-E4), and four indicators related to vaccine policies (V1-V4). The data from the 23 indicators are used to develop five main indices: overall government response index, containment and health index, stringency index, economic support index, and risk of openness index. The stringency index represents the strictness of a government's policy measures taken over time to restrict individual's movements and behaviour. It is calculated based on nine indicators related to containment, closure, and health policies, including: (a) school and university closure, (b) workplace closure, (c) public event cancellations and restrictions, (d) restrictions on private gatherings, (e) public transport closures, (f) stay-at-home orders, (g) restrictions on intra-provincial travel, (h) restrictions on international travel, and (i) public information campaigns. The stringency index is simply the arithmetic mean of the nine indicators. It ranges from 0 to 100, with 100 representing the most stringent response [15, 16].

$$stringency\ index = \frac{1}{N}\sum_{i=1}^{N} Indicator_i \tag{1}$$

where N is the number of indicators used in the index, and $Indicator_i$ is the sub-index score for each indicator. Each sub-index score (Indicator) for any given indicator (i) on any given day (t), is calculated as follows:

$$Indicator_{i,t} = 100\frac{Value_{i,t} - 0.5(Flag_i - BinaryFlag_{j,t})}{MaxValue_i} \tag{2}$$

where $Value_{i,t}$ is the recorded policy value on the ordinal scale. $Flag_i$ is a flag variable corresponds corresponds to the geographic scope of the policy (1 if the policy is general and 0 if targeted). $BinaryFlag_{j,t}$ is the recorded binary flag for the indicator, if the indicator has a flag. $MaxValue_i$ is the maximum value of the indicator [15, 16].

Random forest regression algorithm

Random Forest algorithm is a non-linear statistical ensemble method widely used for both regression and classification. For regression tasks, multiple de-correlated decision trees (N) are constructed at training time, and the mean of the predictions from the trees $(\widehat{Y}(x_i))$ is calculated to produce more consistent and accurate outcomes than using a single tree $(DT_n(x_i))$. To avoid the possibility of over-fitting, a bootstrap sample of the training data (input space) is selected with replacement for every single tree, and a random subset of the available features is selected for splitting (feature space) at each node. The node is then split based on the feature that minimizes the cost function, such as the sum of squared errors. This process continues iteratively until either the predefined maximum depth is reached, or a terminal node is reached. Final predictions are calculated by averaging the results from the individual trees [17, 18].

$$\widehat{Y}(x_i) = \frac{1}{N}\sum_{n=1}^{N} DT_n(x_i) \tag{3}$$

There are several performance metrics to evaluate the predictive accuracy of regression models, but the following four are commonly used: Mean Absolute Error (MAE), Mean Squared Error (MSE), Root Mean Squared Error (RMSE), and Coefficient of Determination (R^2).

$$MAE = \frac{1}{K}\sum_{k=1}^{K} |\widehat{Y}_k - Y_k| \tag{4}$$

$$MSE = \frac{1}{K}\sum_{k=1}^{K} (\widehat{Y}_k - Y_k)^2 \tag{5}$$

$$RMSE = \sqrt{\frac{1}{K}\sum_{k=1}^{K} (\widehat{Y}_k - Y_k)^2} \tag{6}$$

$$R^2 = 1 - \frac{\sum_{k=1}^{K}(\widehat{Y}_k - Y_k)^2}{\sum_{k=1}^{K}(\overline{Y} - Y_k)^2} \tag{7}$$

where K is the number of observations of the training sample, Y_k represents the nth observed value of the dependent variable, $\widehat{Y}_k$ is the nth predicted value of the dependent variable, and $\overline{Y}$ is the average value of the dependent variable.

Bayesian optimization approach

It is necessary to fine-tune the hyperparameters of a particular machine learning model to find its optimal architecture and, therefore, performance. Traditionally, tuning hyperparameters involves setting and testing several combinations of hyperparameters manually. This method, however, becomes inefficient and time-consuming when many hyperparameters are being evaluated. Alternatively, automatic tuning approaches can be used, like grid search, random search, and Bayesian optimization. The Bayesian optimization approach involves manually creating a hyperparameter space from which the algorithm can select a set of hyperparameters for model training. A cross-validation score is then returned by the trained model, and the surrogate function uses the score to recommend new hyperparameters to the objective function that are likely to enhance the performance. This iterative process is repeated until the predefined number of iterations is achieved. Compared with other automatic tuning approaches, this approach typically takes fewer evaluations to find the optimal hyperparameter set [19].

SHAP analysis technique

SHapley Additive exPlanations (SHAP) [20] is a game theoretic approach used to explain the predictions of machine learning models. Specifically, it explains the effect of each feature on the model output and its relative importance based on Shapely values. The Shapely value is calculated by taking the difference between the average model predictions and the predictions of a respective sample. In other words, Shapley values for a specific feature are estimated by comparing the model's predictions with and without that feature. The sign of the Shapely value indicates whether the corresponding feature value has a positive or negative impact on the model's prediction, while magnitude represents how much contribution it makes to prediction. Therefore, the importance of a feature is computed by summing the absolute Shapley values of the feature across the testing dataset samples [21, 22, 23]."

- In "Data analysis and modelling" subsection.

"Data analysis and modelling

Fig 1 demonstrates the methodological framework used in this study. As Fig 1 depicts, the current study involved three main parts. In the first part, we used the actual ridesourcing data to compare the impacts of the COVID-19 pandemic on the ridesourcing services in the Town of Innisfil and

the City of Chicago in terms of the hourly, daily, and monthly distributions of the demand, as well as the origin-destination (OD) flow patterns. In the second part, we explored the impact of the COVID-19 stringency index on the daily and hourly demand values. Moreover, the actual ridesourcing data as well as the COVID-19 related variables and stringency index were used to model the percent reduction in the daily demand for the ridesourcing services in Innisfil and Chicago. In both models, the dependent variable represented the percent reduction in the daily demand values at the town/city level, which were computed based on the average weekday and weekend demand values for the pre-pandemic period (from November 1, 2018 to February 28, 2020).

$$Y_{DR_t} = 100\frac{TD_t - A_{WE} \cdot WE_t - A_{WD} \cdot WD_t}{A_{WE} \cdot WE_t + A_{WD} \cdot WD_t} \tag{8}$$

where Y_{DR_t} is the percent reduction in demand on any given day during the pandemic (t). TD_t is the total number of ridesourcing trips in the town/city. A_{WE} is the pre-pandemic average number of ridesourcing trips on weekends. WE_t is a binary variable representing weekends (1 for weekends and 0 for weekdays). A_{WD} is the pre-pandemic average number of ridesourcing trips on weekdays. WD_t is a binary variable representing weekdays (1 for weekdays and 0 for weekends).

On the other hand, the explanatory variables used in each model included the COVID-19 related variables and stringency index as well as the key trip characteristics, such as: the day-of-week, month-of-year, and weekend dummy variable (to distinguish between weekdays and weekends). In the third part, we modelled the daily direct demand for ridesourcing services in each case study during the pandemic at CT level using COVID-19 related variables and stringency index, land-use data, socioeconomic and demographic characteristics, weather data, and the actual ridesourcing data. In both models, the dependent variable was formed by aggregating trips data, from March 1, 2020 to July 31, 2021 for the City of Chicago and from September 1, 2020 to July 31, 2021 for the Town of Innisfil, at the OD-pair level on each day. Table 1 provides descriptive statistics for both the dependent and explanatory variables.

$$Y_{ij_t} = \sum T_{ij_t} \tag{9}$$

where Y_{ij_t} is the direct demand between two CTs on any given day during the pandemic (t). T_{ij_t} is the total number of ridesourcing trips from CT_i to CT_j.

The percent reduction in the daily demand and the direct demand models were developed using the Random Forest (RF) regression algorithm. In all models, data were randomly divided into training and test sets with 4:1 ratio. Moreover, Bayesian optimization approach was used to find the optimal architecture of each model. The Bayesian optimization approach was implemented using the "fmin" function. We used the Tree Parzen Estimator (TPE) algorithm for the surrogate function, and 65 for the maximum number of iterations. In each iteration, trained models were evaluated using 5-fold cross-validation with the objective function of minimizing the MSE. The best hyperparameter set for each model is presented in S1 Table. The optimal architectures of each model were applied on the testing dataset, and the following performance metrics were computed: MAE, MSE, RMSE, and R^2 (presented in S2 Table). Finally, the SHAP analysis technique was used to interpret the predictions of each model."

Reviewer Point 1.3 — Descriptive statistics of data and commentaries are much needed.

Reply: Thank you for the comment. We have now provided a descriptive statistics for the dependent and explanatory variables in the "Data analysis and modelling" subsection.

- In the "Data analysis and modelling" subsection.

"On the other hand, the explanatory variables used in each model included the COVID-19 related variables and stringency index as well as the key trip characteristics, such as: the day-of-week, month-of-year, and weekend dummy variable (to distinguish between weekdays and weekends). In the third part, we modelled the daily direct demand for ridesourcing services in each case study during the pandemic at CT level using COVID-19 related variables and stringency index, land-use data, socioeconomic and demographic characteristics, weather data, and the actual ridesourcing data. In both models, the dependent variable was formed by aggregating trips data, from March 1, 2020 to July 31, 2021 for the City of Chicago and from September 1, 2020 to July 31, 2021 for the Town of Innisfil, at the OD-pair level on each day. Table 1 provides descriptive statistics for both the dependent and explanatory variables.

$$Y_{ij_t} = \sum T_{ij_t} \tag{10}$$

where Y_{ij_t} is the direct demand between two CTs on any given day during the pandemic (t). T_{ij_t} is the total number of ridesourcing trips from CT_i to CT_j.

Table 1: **Descriptive statistics for the explanatory and dependent variables**

**Variable**	Unit	**Town of Innisfil**				**City of Chicago**			
		Mean	Std. Dev.	Min	Max	Mean	Std. Dev.	Min	Max
Dependent Variables									
Reduction in daily demand	%	28.09	18	0.01	83.61	79.29	15.01	4.58	98.90
Daily OD-pair trips	Count	4	4.32	1	40	2.39	5.34	1	196
Explanatory Variables									
Sociodemographic & socioeconomic									
Median household income	$	60170.9	6987.94	48846.7	68190.72	60489.7	34451.63	11146	194167
Population density	per km^2	433.3	534.94	29.6	1526.1	7183	10335.21	152.5	266431
Percentage of males	%	50	2.11	45.3	51.6	48.3	5.03	25.2	90.3
Percentage of working age group	%	66.69	9.35	45.9	72.7	69.4	8.2	44.1	94.6
Percentage of elderly people	%	17.7	13.03	9	46.7	12.7	6.61	0	53.1
Percentage of married people	%	51.7	4.17	46.5	58	35.2	13.35	0	68.6
Percentage of highly educated people	%	12.6	3.95	8.3	19	36.4	26.3	0.9	94.8
Percentage of personal vehicle commuters	%	10.3	1.94	8.3	13.8	45.1	14.5	10.7	79.6
Average household size	per hhld	2.7	0.33	2	3	2.6	0.58	1.3	4.4
Land use variables									
Residential land use ratio	%	17.5	13.58	1	40	18	9.58	1	52.1
Commercial land use ratio	%	0.02	0.05	0	0.12	3	2.79	0	29.5
Governmental land use ratio	%	0.01	0.04	0	0.1	3.2	5.37	0	42.6
Industrial land use ratio	%	0.21	0.43	0	1.2	2.1	4.74	0	37.8
Parks land use ratio	%	0.9	0.84	0	2.1	0.4	1.94	0	25.3
Weather variables									
Average daily temperature	C	6.8	9.90	−16	25	13.2	10.43	−17	30
Precipitation	mm	0.2	0.52	0	3.51	0.2	0.72	0	9.32
Snowfall	cm	1.4	6	0	40.6	0.3	2.22	0	40.4
COVID-19 related variables & NPIs									
Cases	Count	3.5	3.77	0	19	558	572.49	0	3360
Deaths	Count	0.1	0.25	0	1	10.9	11.80	0	58
Hospitalization	Count	0.5	0.69	0	3	55.7	46	1	203
Positive rate	%	2	1.28	0	5	8.2	7.43	0	33
Percentage of partially vaccinated	%	21.3	28.2	0	77.28	13.96	21.29	0	58.7
Percentage of fully vaccinated	%	7.5	15.08	0	64	10.7	18	0	52.3
Stringency index	%	68.8	15.4	41.7	90.7	56.8	14.49	5.6	82.4
Trip related variables									
Day of the week	–	–	–	1	7	–	–	1	7
Weekend	–	–	–	0	1	–	–	0	1
Month of the year	–	–	–	1	12	–	–	1	12
year	–	–	–	0	1	–	–	0	1

The dependent variables, weather variables, and COVID-19 related variables and NPIs statistics were calculated based on the period September 1, 2020, to July 31, 2021, for the Town of Innisfil,

and March 1, 2020, to July 31, 2021, for the City of Chicago. Percentage of highly educated people variable represents the percentage of population with at least an undergraduate degree. Stringency index values are for Ontario and Illinois. Day of the week is a discrete variable represents the day of the week from Monday to Sunday. Weekend is a binary variable to distinguish between weekends and weekdays, with 1 for weekends. Month of the year is a discrete variable represents the month of the year from January to December. Year is a binary variable to distinguish between 2020 and 2021, with 1 for 2021."

Reviewer Point 1.4 — If the term "post-pandemic" was used, the authors should clearly state that which period was considered as post-pandemic.

Reply: Thank you for the comment. "Post-pandemic" refers to the period following the declaration of COVID-19 as a global pandemic in this study. We have now defined the term in the Introduction Section following its first use.

"In the current study, COVID-19 related variables and NPIs, land-use data, socioeconomic and demographic characteristics, weather data, and the actual ridesourcing data for the City of Chicago, Illinois, and the Town of Innisfil, Ontario, from November 2018 to August 2021 are used to analyze and compare (a) the impacts of the COVID-19 pandemic on the spatio-temporal patterns of the demand, (b) the effects of the severity of the NPIs, COVID-19 related variables, and vaccination rates on the percent reduction in the daily demand, as well as (c) the main factors affecting the direct demand (origin-destination-pair) in the post-pandemic era (the period following the declaration of COVID-19 as a global pandemic).".

Reviewer Point 1.5 — Would modeling the reduction of OD-pair trips gain more insights?

Reply: Thank you for the comment. We agree that modelling the reduction in the demand at the OD-pair level would provide more insights than modelling at the town/city level. However, the data we would need to develop such models are not available for both case studies, as pre-pandemic data for Innisfil are only available at the aggregate level (town level). Thus, for consistency and accuracy, we modelled the daily reduction in demand at the town and city level.

Reviewer Point 1.6 — Discussion should be improved. For example, how did the results link to area's unique characteristics? How the transport policy or plan could be developed based on the findings? Any transportation policy implications with regards to Covid restrictions?

Reply: Thank you for the comment. This section has been extensively revised to discuss the results and relate them to the characteristics of each case study, and to provide useful policy recommendations for planners and service providers.

"Discussion and concluding remarks

The on-going COVID-19 pandemic has significantly impacted individual's mobility [24, 25]. Understanding these effects can help planners and policy makers to better anticipate the pandemic's future impacts, which may contribute both to recovery efforts and plan responses to additional waves of COVID-19 or other pandemics. Previous studies, however, have only considered the impacts of COVID-19 on ridesourcing services in early stages and have primarily focused on large cities. This is mainly due to the apparent lack of readily available ridesourcing data. In this study, we examined and compared the impact of the COVID-19 pandemic on ridesourcing services in small towns and large cities using actual data from the Town of Innisfil, Ontario, and the City of Chicago, Illinois. The main factors that governed the selection of these case studies were: a) the availability of ridesourcing data, b) the coverage of both the pre-pandemic and pandemic periods, and c) the location of both case studies within the same region (North America). The two case studies are located in North America, have similar weather conditions, share some sociodemographic characteristics, such as percentage of males, percentage of working age group, and average household size, and have experienced various NPIs during the pandemic. On the flip side, they differ in the land area, population density, the use of ridesourcing services and their operating policies, as well as the severity of NPIs over time.

Overall, the pandemic has affected the usage behaviour of ridesourcing services in both Innisfil and Chicago. The ridesourcing demand was most affected at night and on weekends, as a consequence of closing non-essential businesses. The physical and mental health issues associated with the pandemic resulted in fewer trips in areas with higher proportions of seniors and more trips to parks and green spaces. Moreover, the results revealed that the demand was adversely affected by the stringency index and COVID-19-related variables, and positively affected by vaccination rates. However, the extent of the effects of pandemic-related factors varied across the two case studies. First, the demand in Innisfil, where ridesourcing services are subsidized and used mainly for work-related purposes, experienced a markedly lower reduction rate, was less affected by the increase in the stringency index, and showed higher recovery rates following each relaxation of restrictions than in Chicago. Second, the low daily number of hospitalizations and deaths (maximum daily values were at 1 and 3, respectively) from COVID-19 in Innisfil, resulted in these factors having the least impact on individuals' use of the service compared with other COVID-19 related variables. In contrast, the positive rate variable, which indicates the level of COVID-19 transmission in the community, had the highest impact on individuals' demand for the service, most likely because it increased their fear of getting infected. The opposite pattern was observed in Chicago, where the COVID-19 hospitalization and death rates were substantially higher than in Innisfil (maximum daily values were at 203 and 58, respectively). The high number of hospitalizations and deaths probably increased individuals' concerns about the consequences of the virus, and, therefore, affected their use of the service most, compared with other COVID-19 variables. Last, compared with Chicago, vaccination rates had a higher impact on the demand for ridesourcing services in Innisfil. It is possible that the low population density in Innisfil, along with the low number of COVID-19 cases, hospitalizations, and deaths, resulted in more people feeling comfortable participating in different activities after receiving the vaccination.

We provide the following recommendations for planners, policymakers, and service providers, which can be useful in recovering ridesourcing services and planning for additional COVID-19 waves or other pandemics:

- *Previous literature revealed that public transit services have experienced a significant reduction in demand during the early stages of the pandemic [26, 27]. Governments' policy measures during this period, such as stay at home orders and reduced capacity policies, may have affected the efficiency, operating costs, and performance of public transit services, particularly in low density areas. In light of this, we recommend subsidizing ridesourcing services in the low-density areas rather than operating the existing fixed-route public transit system at high stringency levels. The use of these services requires no capital expenditures and can help limit individuals' interaction. It can also provide essential workers with flexible and convenient transportation, create new jobs for those who lost their jobs due to the pandemic, and support service providers. This suggestion, however, needs further study to verify its feasibility and validity.*

- *Our results indicated that COVID-19 variables adversely affect individual demand for ridesourcing services. Therefore, transportation service providers should ensure that drivers and passengers follow the essential COVID-19 preventative measures to to restore individuals' confidence in the safety of ridesourcing services and foster the recovery of the service. Providing users with the opportunity to rate the driver's preventative measures might be useful.*

- *Our findings revealed that ridesourcing services experienced a repeated pattern of a sharp decrease in demand followed by a gradual recovery. It is possible that this has impacted the number of drivers and their working hours. Thus, transportation service providers should provide a proper incentive strategy for drivers to ensure that there is adequate supply as the demand recovers and that performance remains the same as it was before the pandemic. The demand models obtained in this work might be of use for the service providers in Chicago and Innisfil to anticipate the demand as the policy measures change. Although the models may not be suitable for ridesourcing services in other locations, they can provide key insights about the factors affecting the demand.*

- *The use of the pandemic-related factors contributed to interpreting the temporal variations in demand for ridesourcing and understanding their effects. This suggests the importance of incorporating them in future travel behaviour studies.*

This research has two main limitations: (a) Innisfil's ridesourcing dataset does not cover the early stages of the pandemic (from March to August 2020), and (b) our findings are based on ridesourcing data from Innisfil and Chicago, therefore, they may not be directly generalized to other towns or cities. Future research may explore the impact of the COVID-19 pandemic on the demand for other modes of transportation as well as the supply of ridesourcing services. Another future direction could be to investigate the factors affecting the recovery of public transit and ridesourcing services."

Reviewer Point 1.7 — Figs 2 and 3 are probably switched.

Reply: Thank you for letting us know. The figures are now arranged correctly.

Reviewer 2

This paper aims to investigate the effect of COVID-19 on RS demand. This manuscript is fascinating, and I am impressed with this; however, I have some suggestions that the authors need to act on.

Reply: We would like to thank the reviewer for the time, as well as the constructive comments and suggestions. Point-by-point responses to the comments are presented below.

Reviewer Point 2.1 — Abstract:
The authors stated that "it is still unclear how the pandemic affected the demand for ridesourcing Services". In fact, some studies show that the demand for RS decrease due to pandemic (for example, see: https://doi.org/10.1007/s11116-021-10185-5. Due to this, the authors need to revise that sentence.

Reply: Thank you for your comment. We agree that previous studies have examined some aspects of the COVID-19 pandemic's impact on ridesourcing services, but they have not covered all of them. The topic needs to be explored from several perspectives to have a complete understanding of how the pandemic affected the service. These include questions like, did the pandemic effects on ridesourcing services vary temporally and spatially? and why? What factors affect the recovery of the service? What effects has the pandemic had on individual's attitudes and perceptions towards the service? and for how long these effects will last?, and more. Thus, we believe this sentence is still valid, but in order to be more precise, we have modified the first two sentences of the abstract as follows:
"To curb the spread of the ongoing 2019 novel coronavirus (COVID-19), authorities have adopted several non-pharmaceutical (NPIs) and pharmaceutical interventions, which significantly affected our daily activities and mobility patterns. However, it is still unclear how severity of NPIs, COVID-19-related variables, and vaccination rates have affected demand for ridesourcing services, and whether these effects vary across small towns and large cities.".

Reviewer Point 2.2 — Introduction:
1. "Previous literature revealed that the pandemic has significantly influenced all modes of transportation" *use and frequency of use (please add references)

Reply: Thank you for the comment. The sentence has been updated and references have been added. The first sentence in the second paragraph of the Introduction is now as follows:
"Previous literature revealed that the pandemic has significantly influenced demand for all modes of transportation [3, 4] as well as changed individual's travel behaviour and preferences [6, 7, 8, 9]"

Reviewer Point 2.3 — Introduction:
2. Data collected from November 2018 to August 2021. Explain a brief of government policies were applied to suppress the COVID-19 during the period in two cities. Clearly, it significantly influences the number of trips. I read that the authors have explained it in lines 122-128. But please explain in more detail because I believe that the government policies during the study period are changing rapidly.

Reply: Thank you for the comment. We have a brief of the policy measures taken by Ontario and Illinois during the study period in the "Data collection" subsection.

- In the "Data collection" subsection:

"In this study, data from multiple sources were utilized to examine this hypothesis. Weather data were collected from AccuWeather Inc. [5] for both case studies. The collected dataset contained information on the daily average temperature, precipitation, and snowfall for the period of March 1, 2020 to July 31, 2021. Socioeconomic and demographic factors were obtained from the 2016 Statistics Canada and the 2017 American Community Survey for Innisfil and Chicago, respectively. Several characteristics were collected for both case studies at the census tract level (CT), including population density, median income, gender (percentage of males), percentage of elderly people, education, marital status, and mode of commuting. Land-use data were obtained from the Chicago Metropolitan Agency for Planning and the Town of Innisfil. The COVID-19 related variables were acquired from the Simcoe Muskoka District Health Unit and Chicago COVID Dashboard for the period of January 1, 2020 to July 31, 2021. The main COVID-19 related variables considered in this study were the daily confirmed cases, deaths, hospitalizations, test positivity rate (percentage of tests performed that were positive for COVID-19), as well as the cumulative percentage of residents who received the first and second vaccine doses. As for the COVID-19 NPIs, several interventions have been implemented in Ontario and Illinois since the beginning of the pandemic, including the declaration of a state of emergency, closing schools, restaurants, and bars. Since the beginning of the pandemic, Ontario has declared the state of emergency (stay at home order) three times. The first state of emergency was declared on March 17, 2020, and ended on July 24, 2020. On January 14, 2021, a second state of emergency was announced, and it ended on February 19, 2021. The last state of emergency was declared on April 8, 2021, and ended on June 9, 2021. These states of emergency were preceded by the closure of schools, restaurants, bars, and non-essential workplaces [13]. On the other hand, Illinois declared only one state of emergency on March 21, 2020, which lasted until May 29, 2020. The state of emergency was followed by the reopening of schools and relaxation of most COVID-19-related measures by the end of June. Then several cycles of restaurant and bar closures and reopenings took place between the period of August 2020 and May 2021. In May 2021, fully vaccinated people were exempted from wearing mast for most indoor activities [14]. We used used Stringency Index data from the Oxford COVID-19 Government Response Tracker (OxCGRT)[15] to represent the severity of the government's policy measures in both locations. The data was collected from January 1, 2020, to July 31, 2021, for both case studies."

Reviewer Point 2.4 — Materials and Methods: Data collection
1. The authors stated that data for pre-pandemic period in Innisfil were obtained

from Uber Technologies. Are there any other RS companies instead of Uber in that city? If yes, it could make the biased data. The authors need to explain about this.

Reply: Thank you for bringing this to our attention. In fact, Uber Technologies is the only transportation network provider in Innisfil. This is now mentioned in the Data Collection section. *"In May 2017, the Town of Innisfil partnered with Uber Technologies Inc., the only transportation network provider (TNP) in the town, to provide residents with subsidized ridesourcing trips rather than operate a fixed-route transit system [10, 11]. Therefore, the service has been used for a wide variety of trip purposes including work, school, shopping, social, recreation, and medical appointments [12]."*.

Reviewer Point 2.5 — Materials and Methods: Data collection
2. Line 141-145: make it a sentence

Reply: Thank you for the comment. We now present the time periods as follows: *"Moreover, the average hourly demand data were provided for five time periods: morning (6:00 AM to 10:00 AM), midday (10:00 AM to 3:00 PM), late afternoon (3:00 PM to 7:00 PM), evening (7:00 PM to 10:00 PM), and night (10:00 PM to 6:00 AM)."*.

Reviewer Point 2.6 — Results
1. The authors stated that they use RF regression algorithms and include four performance metrics to evaluate the predictive accuracy of the models. However, I can not see those performance metrics in the result section. How can the authors ensure the readers and me that their model is correct?

Reply: Thank you for the comment. The optimal hyperparameters as well as the performance metrics of the developed models are provided in the "Supporting information" section. We have referred to these tables in the last paragraph of the "Data analysis and modelling" section.

We have used several ways to ensure the quality of the modelling results.

- Providing a detailed methodology for data collection, analysis, and modelling. We have also explained the techniques as well as the technical measurements used in the modelling part in the revised version.
- Using a powerful automatic tuning approach (Bayesian Optimization Method) for the hyperparameters of the Random Forest algorithm, which ensures that each model used the hyperparameters that produced the best performance.
- Providing the key performance metrics for each model.
- Using the SHAP analysis technique to interpret the predictions of each model.
- Providing the code used to produce the models presented in this study along with the minimal data.

In the "Data analysis and modelling" section.

"The percent reduction in the daily demand and the direct demand models were developed using the Random Forest (RF) regression algorithm. In all models, data were randomly divided into training and test sets with 4:1 ratio. Moreover, Bayesian optimization approach was used to find the optimal architecture of each model. The Bayesian optimization approach was implemented using the "fmin" function. We used the Tree Parzen Estimator (TPE) algorithm for the surrogate function, and 65 for the maximum number of iterations. In each iteration, trained models were evaluated using 5-fold cross-validation with the objective function of minimizing the MSE. The best hyperparameter set for each model is presented in S1 Table. The optimal architectures of each model were applied on the testing dataset, and the following performance metrics were computed: MAE, MSE, RMSE, and R^2 (presented in S2 Table). Finally, the SHAP analysis technique was used to interpret the predictions of each model."

Reviewer Point 2.7 — Results:
2. The authors need to explain how to understand whether the considered variables significantly influence RH use or not. If I check your discussion, all included variables in Fig. 6 significantly influenced RH use with positive and negative signs. When I use the discrete choice model, for example, each variable has a p-value to determine whether or not a variable is significant.

Reply: Thank you for the comment. SHAP analysis results do not provide insight into whether an explanatory variable impacts the model outcomes significantly, but rather explain its effects and relative importance compared to other explanatory variables. We have now provided more details about the techniques used in the modelling part, including SHAP analysis method, under the "Methods" subsection of the "Materials and methods" section.

- In the "Methods" subsection.

"SHAP analysis technique

SHapley Additive exPlanations (SHAP) [20] is a game theoretic approach used to explain the predictions of machine learning models. Specifically, it explains the effect of each feature on the model output and its relative importance based on Shapely values. The Shapely value is calculated by taking the difference between the average model predictions and the predictions of a respective sample. In other words, Shapley values for a specific feature are estimated by comparing the model's predictions with and without that feature. The sign of the Shapely value indicates whether the corresponding feature value has a positive or negative impact on the model's prediction, while magnitude represents how much contribution it makes to prediction. Therefore, the importance of a feature is computed by summing the absolute Shapley values of the feature across the testing dataset samples [21, 22, 23]."

Reviewer Point 2.8 — Results:
3. How can the authors state that a variable has the highest effect while other variables

have the lowest impact? Does it depend on the values in Fig. 6.? If yes, the authors need to state it.

Reply: Thank you for the comment. The position of an explanatory variable on the vertical axis indicates its relative importance for the model outcomes compared to other explanatory variables. We provided a description of the SHAP summary plot in the caption of Fig 5, where it first appeared in the manuscript. Moreover, we have now added a legend to both Figs 5 and 6 to make it easier for readers to understand these plots.

- In the caption of Fig 5:

"Each point in the summary plot represents a Shapley value for a feature and an instance. The feature importance determines its position on the y-axis, the Shapley value determines its position on the x-axis, and the value determines its colour."

Reviewer Point 2.9 — Discussion and concluding remarks
1. Delete lines 389-394, because you have clearly stated it in the introduction section.
2. More importantly, clearly state how your findings fill the gap of previous findings.
3. The authors mentioned that the study contributes to policymakers as well as service providers to understand the future impact of the pandemic on ridesourcing services in terms of the recovery of the service, preparedness for additional waves of COVID-19 as well as other pandemics. Please add some examples of government policies in the discussion section based on your research findings to strengthen your statements.

Reply: Thank you for the comment. This section has been extensively revised. We have removed lines 389-394, discussed the results and linked them to the characteristics of each case study, explained how our study fill the gap in the literature, and provided useful policy recommendations for planners and service providers.

"Discussion and concluding remarks

The on-going COVID-19 pandemic has significantly impacted individual's mobility [24, 25]. Understanding these effects can help planners and policy makers to better anticipate the pandemic's future impacts, which may contribute both to recovery efforts and plan responses to additional waves of COVID-19 or other pandemics. Previous studies, however, have only considered the impacts of COVID-19 on ridesourcing services in early stages and have primarily focused on large cities. This is mainly due to the apparent lack of readily available ridesourcing data. In this study, we examined and compared the impact of the COVID-19 pandemic on ridesourcing services in small towns and large cities using actual data from the Town of Innisfil, Ontario, and the City of Chicago, Illinois. The main factors that governed the selection of these case studies were: a) the availability of ridesourcing data, b) the coverage of both the pre-pandemic and pandemic periods, and c) the location of both case studies within the same region (North America). The two case studies are located in North America, have similar weather conditions, share some sociodemographic charac

teristics, such as percentage of males, percentage of working age group, and average household size, and have experienced various NPIs during the pandemic. On the flip side, they differ in the land area, population density, the use of ridesourcing services and their operating policies, as well as the severity of NPIs over time.

Overall, the pandemic has affected the usage behaviour of ridesourcing services in both Innisfil and Chicago. The ridesourcing demand was most affected at night and on weekends, as a consequence of closing non-essential businesses. The physical and mental health issues associated with the pandemic resulted in fewer trips in areas with higher proportions of seniors and more trips to parks and green spaces. Moreover, the results revealed that the demand was adversely affected by the stringency index and COVID-19-related variables, and positively affected by vaccination rates. However, the extent of the effects of pandemic-related factors varied across the two case studies. First, the demand in Innisfil, where ridesourcing services are subsidized and used mainly for work-related purposes, experienced a markedly lower reduction rate, was less affected by the increase in the stringency index, and showed higher recovery rates following each relaxation of restrictions than in Chicago. Second, the low daily number of hospitalizations and deaths (maximum daily values were at 1 and 3, respectively) from COVID-19 in Innisfil, resulted in these factors having the least impact on individuals' use of the service compared with other COVID-19 related variables. In contrast, the positive rate variable, which indicates the level of COVID-19 transmission in the community, had the highest impact on individuals' demand for the service, most likely because it increased their fear of getting infected. The opposite pattern was observed in Chicago, where the COVID-19 hospitalization and death rates were substantially higher than in Innisfil (maximum daily values were at 203 and 58, respectively). The high number of hospitalizations and deaths probably increased individuals' concerns about the consequences of the virus, and, therefore, affected their use of the service most, compared with other COVID-19 variables. Last, compared with Chicago, vaccination rates had a higher impact on the demand for ridesourcing services in Innisfil. It is possible that the low population density in Innisfil, along with the low number of COVID-19 cases, hospitalizations, and deaths, resulted in more people feeling comfortable participating in different activities after receiving the vaccination.

We provide the following recommendations for planners, policymakers, and service providers, which can be useful in recovering ridesourcing services and planning for additional COVID-19 waves or other pandemics:

- *Previous literature revealed that public transit services have experienced a significant reduction in demand during the early stages of the pandemic [26, 27]. Governments' policy measures during this period, such as stay at home orders and reduced capacity policies, may have affected the efficiency, operating costs, and performance of public transit services, particularly in low density areas. In light of this, we recommend subsidizing ridesourcing services in the low-density areas rather than operating the existing fixed-route public transit system at high stringency levels. The use of these services requires no capital expenditures and can help limit individuals' interaction. It can also provide essential workers with flexible and convenient transportation, create new jobs for those who lost their jobs due to the pandemic, and support service providers. This suggestion, however, needs further study to verify its feasibility and validity.*

- *Our results indicated that COVID-19 variables adversely affect individual demand for rides*

ourcing services. Therefore, transportation service providers should ensure that drivers and passengers follow the essential COVID-19 preventative measures to restore individuals' confidence in the safety of ridesourcing services and foster the recovery of the service. Providing users with the opportunity to rate the driver's preventative measures might be useful.

- *Our findings revealed that ridesourcing services experienced a repeated pattern of a sharp decrease in demand followed by a gradual recovery. It is possible that this has impacted the number of drivers and their working hours. Thus, transportation service providers should provide a proper incentive strategy for drivers to ensure that there is adequate supply as the demand recovers and that performance remains the same as it was before the pandemic. The demand models obtained in this work might be of use for the service providers in Chicago and Innisfil to anticipate the demand as the policy measures change. Although the models may not be suitable for ridesourcing services in other locations, they can provide key insights about the factors affecting the demand.*

- *The use of the pandemic-related factors contributed to interpreting the temporal variations in demand for ridesourcing and understanding their effects. This suggests the importance of incorporating them in future travel behaviour studies.*

This research has two main limitations: (a) Innisfil's ridesourcing dataset does not cover the early stages of the pandemic (from March to August 2020), and (b) our findings are based on ridesourcing data from Innisfil and Chicago, therefore, they may not be directly generalized to other towns or cities. Future research may explore the impact of the COVID-19 pandemic on the demand for other modes of transportation as well as the supply of ridesourcing services. Another future direction could be to investigate the factors affecting the recovery of public transit and ridesourcing services."

References

1. Canada S. Population and dwelling count highlight tables, 2016 census. Statistics Canada Ottawa, ON, Canada. 2017.

2. American CSO. Data Profile. The United States Census Bureau. 2017.

3. Bucsky, Péter Modal share changes due to COVID-19: The case of Budapest. Transportation Research Interdisciplinary Perspectives. 2020 Nov 1;8:100141.

4. Yu J, Xie N, Zhu J, Qian Y, Zheng S, Chen XM. Exploring impacts of COVID-19 on city-wide taxi and ride-sourcing markets: Evidence from Ningbo, China. Transport Policy. 2022 Jan 1;115:220-38.

5. Local, National, & Global Daily Weather Forecast. AccuWeather. 2021.

6. Dingil AE, Esztergár-Kiss D. The Influence of the Covid-19 Pandemic on Mobility Patterns: The First Wave's Results. Transportation Letters. 2021 May 28;13(5-6):434-46.

7. Abdullah M, Dias C, Muley D, Shahin M. Exploring the impacts of COVID-19 on travel behavior and mode preferences. Transportation research interdisciplinary perspectives. 2020 Nov 1;8:100255.

8. Yang Y, Cao M, Cheng L, Zhai K, Zhao X, De Vos J. Exploring the relationship between the COVID-19 pandemic and changes in travel behaviour: A qualitative study. Transportation Research Interdisciplinary Perspectives. 2021 Sep 1;11:100450.

9. Morshed SA, Khan SS, Tanvir RB, Nur S. Impact of COVID-19 pandemic on ride-hailing services based on large-scale Twitter data analysis. Journal of Urban Management. 2021 Jun 1;10(2):155-65.

10. Cane T. Demand-Responsive Transit Implementation - Stage 1. Publication DSR-042-17. Innisfil, ON: Town of Innisfil Report; 2017.

11. Sanaullah I, Alsaleh N, Djavadian S, Farooq B. Spatio-temporal analysis of on-demand transit: A case study of Belleville, Canada. Transportation Research Part A: Policy and Practice. 2021 Mar 1;145:284-301.

12. Matthias S., Raktim M., Sarin C. Innisfil Transit and Social Outcomes. Ryerson University, TransForm Lab. 2020.

13. Covid-19 intervention timeline in Canada. Canadian Institute for Health Information. 2022 June 09.

14. Documenting Illinois' path to recovery from the coronavirus (COVID-19) pandemic, 2020-2021. Ballotpedia. 2022.

15. Hale T, Angrist N, Goldszmidt R, Kira B, Petherick A, Phillips T, Webster S, Cameron-Blake E, Hallas L, Majumdar S, Tatlow H. A global panel database of pandemic policies (Oxford COVID-19 Government Response Tracker). Nature Human Behaviour. 2021 Apr;5(4):529-38.

16. Petherick A, Kira B, Hale T, Phillips T, Webster S, Cameron-Blake E, et al. Variation in government responses to COVID-19. Blavatnik Centre for Government Working Paper, University of Oxford. 2020 Sep 1.

17. Breiman L. Random forests. Machine learning. 2001 Oct;45(1):5-32.

18. Hutengs C, Vohland M. Downscaling land surface temperatures at regional scales with random forest regression. Remote Sensing of Environment. 2016 Jun 1;178:127-41.

19. Yang L, Shami A. On hyperparameter optimization of machine learning algorithms: Theory and practice. Neurocomputing. 2020 Nov 20;415:295-316.

20. Lundberg SM, Lee SI. A unified approach to interpreting model predictions. Advances in neural information processing systems. 2017;30.

21. Alsaleh N, Farooq B. Interpretable data-driven demand modelling for on-demand transit services. Transportation Research Part A: Policy and Practice. 2021 Dec 1;154:1-22.

22. Kalatian A, Farooq B. Decoding pedestrian and automated vehicle interactions using immersive virtual reality and interpretable deep learning. Transportation research part C: emerging technologies. 2021 Mar 1;124:102962.

23. Twick I., Zahavi G., Benvenisti H., Rubinstein R., Woods MS., Berkenstadt H., et al. Towards interpretable, medically grounded, EMR-based risk prediction models. Scientific reports. 2022 Jun 15;12(1):1-2.

24. Wellenius GA, Vispute S, Espinosa V, Fabrikant A, Tsai TC, Hennessy J, Dai A, Williams B, Gadepalli K, Boulanger A, Pearce A. Impacts of social distancing policies on mobility and COVID-19 case growth in the US. Nature communications. 2021 May 25;12(1):1-7.

25. Chan HF, Skali A, Savage DA, Stadelmann D, Torgler B. Risk attitudes and human mobility during the COVID-19 pandemic. Scientific reports. 2020 Nov 16;10(1):1-3.

26. Rothengatter W, Zhang J, Hayashi Y, Nosach A, Wang K, Oum TH. Pandemic waves and the time after Covid-19–Consequences for the transport sector. Transport Policy. 2021 Sep 1;110:225-37.

27. Liu L, Miller HJ, Scheff J. The impacts of COVID-19 pandemic on public transit demand in the United States. Plos one. 2020 Nov 18;15(11):e0242476.
